# FunBlocks. A Modular Framework for AmI System Development

**DOI:** 10.3390/s120810259

**Published:** 2012-07-30

**Authors:** Rafael Baquero, José Rodríguez, Sonia Mendoza, Dominique Decouchant, Alfredo Piero Mateos Papis

**Affiliations:** 1 Department of Computer Science, CINVESTAV-IPN, Instituto Politécnico Nacional 2508, San Pedro Zacatenco, Del. Gustavo A. Madero, DF 07360, Mexico; E-Mails: rodriguez@cs.cinvestav.mx (J.R.); smendoza@cs.cinvestav.mx (S.M.); 2 Department of Information Technologies, UAM Cuajimalpa, Av. Constituyentes 1000 Lomas Altas, Del. Miguel Hidalgo, DF 11950, Mexico; E-Mails: decouchant@correo.cua.uam.mx (D.D.); amateos@correo.cua.uam.mx (A.P.M.P.); 3 Centre National de la Recherche Scientifique, Laboratoire LIG, 681 Rue de la Passerelle, 38400 St Martin d'Hères, France

**Keywords:** Ambient Intelligence, AmI frameworks, modular schemes, distributed control systems, domotics

## Abstract

The last decade has seen explosive growth in the technologies required to implement Ambient Intelligence (AmI) systems. Technologies such as facial and speech recognition, home networks, household cleaning robots, to name a few, have become commonplace. However, due to the multidisciplinary nature of AmI systems and the distinct requirements of different user groups, integrating these developments into full-scale systems is not an easy task. In this paper we propose FunBlocks, a minimalist modular framework for the development of AmI systems based on the function module abstraction used in the IEC 61499 standard for distributed control systems. FunBlocks provides a framework for the development of AmI systems through the integration of modules loosely joined by means of an event-driven middleware and a module and sensor/actuator catalog. The modular design of the FunBlocks framework allows the development of AmI systems which can be customized to a wide variety of usage scenarios.

## 1. Introduction

Ambient Intelligence (AmI) is a vision of smart environments that are reactive to people and able to make our actions safer, more efficient, more informed, more comfortable or simply more enticing. In this vision our environments will be embedded with different types of sensing systems, pervasive devices, and networks that can perceive and react to people [1]. Ambient Intelligence has been described by researchers in different ways, but two characteristics that an AmI system must possess are to be sensitive and responsive [2]. Through sensors, AmI systems gather information about the environment, process that information in some manner and finally through actuators modify the environment in a form that will benefit users in the environment [3].

A control system is a device or set of devices to manage, command, direct or regulate the behavior of other devices or systems [4]. As such, an AmI system can be viewed as a type of control system. Furthermore, AmI systems can be viewed as an evolution of Home Automation (HA) and Building Automation (BA) systems.

Despite the many advantages for everyday living offered by AmI and HA technologies there has not been a widespread offer or demand of AmI or HA products. Although HA has been available to consumers since the 1970s, it has not been adopted in such a wide scale as, for example, cellular phones. Probably the main reason why HA technology has not enjoyed wider demand are the difficulties users encounter when trying to bring this technology to their daily lives and the lack of a standardized means of interaction with the devices. In [5] Bernheim *et al.* surveyed 14 households which employed HA. They found four major challenges people face when deploying and using such systems: high cost of ownership, inflexibility, poor manageability, and difficulty achieving security. In other words, HA systems are not currently “user friendly”.

Current AmI systems are also not “researcher friendly”. After more than a decade of considerable research the field has not yet matured to the point of enabling incremental research. Much of the research effort still seems devoted to the creation, very often from scratch, of new technologies and systems [1]. Ambient Intelligence is a highly multi-disciplinary field which involves communications, control systems, electronics, artificial intelligence, human-computer interfaces, distributed systems and others. However there is not a common set of tools which researchers from different disciplines can use to contribute to the field of AmI research.

To address these issues we are developing FunBlocks, a minimalist modular framework for the development of AmI systems. FunBlocks is based on the function module abstraction used in the IEC 61499 standard for distributed control systems. Through the use of function modules FunBlocks promotes component reuse which allows researchers from different fields to easily apply previous results in new developments.

FunBlocks is also targeted at the development of commercially viable AmI systems. Using FunBlocks system integrators can develop highly customizable AmI systems where end users can add new functions with a degree of difficulty analogous to hooking up a common home appliance or installing a new program on a computer.

As mentioned previously, AmI systems require the use of sensors and actuators both to obtain information about the environment and to adjust this environment to the user's needs. There are many different sensor and actuator communications protocols available for AmI systems. It is a desirable feature for an AmI system to be able to handle many different types of sensor and actuator communication protocols since this provides added flexibility to the system and also because in many instances such sensors and actuators may already be installed in a given environment. To provide a rough idea of the differences in currently available sensor and actuator communication protocols, in Section 2 we provide a brief description of some of these protocols.

Building Automation (BA) and Home Automation (HA) products have been available for several years. Although not in widespread use in personal residences, there are many BA systems currently installed in modern office buildings. Since the investment in BA systems can be relatively high, it would be unreasonable to expect those systems to be discarded and simply replaced by new AmI systems. As a result AmI systems will have to coexist and interact with existing BA and HA systems. In Section 3 we provide a brief description of some of the most commonly used communication standards in the building automation industry.

The development of frameworks for AmI and related fields, such as AAL and UbiComp, has been an area of intense research in the last years. As a result several frameworks have been proposed. In Section 4 we provide a brief description of some of these frameworks.

To provide some background on the use of function modules in distributed control systems, the fundamental concepts of IEC 61499 are described in Section 5. In Section 6 we describe the FunBlocks framework and a brief features comparison of the frameworks described is provided in Section 7. Finally, in Section 8, we describe future work that we have planned for the development of FunBlocks.

## 2. Sensor and Actuator Communications Protocols

A sensor is a device that receives a stimulus and responds with an electrical signal. The purpose of a sensor is to respond to some kind of an input physical property (stimulus) and to convert it into an electrical signal that is compatible with electronic circuits [6]. There is a wide array of communication protocols developed for use with sensors and actuators. In this section we describe some commonly used protocols.

### 2.1. X-10

X-10 is a domotics communication standard introduced in the 1970s. The original X-10 protocol was intended to control devices and thus did not incorporate any means of receiving data from sensors. The protocol was later expanded with the introduction of extended commands to allow its use in sensors. Due to the low cost and wide availability of X-10 based devices this standard still enjoys widespread use.

X-10 is a power line based communication standard which employs 120 kHz bursts synchronized with the zero crossing of the power line's alternating current waveform. A binary ‘1’ is represented by a 1 ms burst near the zero crossing while a binary ‘0’ is represented by the absence of such a burst [7].

Standard X-10 messages are composed by a Start Code, followed by a House Code and finally a Key Code. The Start Code is represented by the unique sequence ‘1110’, while House Codes and Key Codes employ complementary pairs for each bit.

The House Code is a 4-bit identifier intended to avoid commands destined for one house from activating sensors or actuators in another house. The 5-bit Key Code can either indicate the address of an X-10 module, a standard command, or can indicate the beginning of an extended command. Addresses and commands are distinguished from each other by means of the Key Code's LSB. If the LSB is ‘1’ then the Key Code in question is a command, while if the LSB is a ‘0’ the Key Code is an address. When an X-10 module is addressed through an appropriate Key Code, it will continue to respond to X-10 commands until either a new address is issued or an “All Units Off” command is issued [8].

Standard commands are X-10 commands which allow a system to interact with actuators and perform functions such as turning lights or devices on or off, but do not provide a means to obtain readings from sensors. Obtaining data from sensors or sending more complex commands to actuators requires the use of extended commands. After the beginning of an extended command is signaled by an appropriate Key Code, the rest of the extended command is made up of a 4-bit address, an 8-bit data field and an 8-bit command.

In order to avoid collisions, before using the medium an X-10 transmitter must wait for a random interval between 8 and 10 zero-crossings. If during this interval there have not been any data ‘1’ bits transmitted, the device can begin transmission. During the transmission of each ‘0’ bit (no 120 kHz burst) the transmitter must monitor the medium for 120 KHz bursts to detect a collision. If a collision is detected, the transmitter must abort its transmission and recommence the transmission process.

### 2.2. IEEE 802.15.4

IEEE 802.15.4 wireless technology is a short-range communication system intended for Low-Rate Wireless Personal Areas Networks (WPANs) [9]. The key features of the IEEE 802.15.4 wireless technology are low complexity, low cost, low power consumption and low data rate transmissions to be supported by cheap, fixed or moving devices [10]. The IEEE 802.15.4 standard provides specifications for the MAC and PHY layers. For a definition of the upper layers other standards, such as the ZigBee stack specified by the industrial consortia ZigBee Alliance [11] and the IPv6 over Low-power PAN (6LowPAN) [12], have been developed. The IEEE 802.15.4 PHY operates using Direct Sequence Spread Spectrum in three different unlicensed bands according to the geographical area where the system is deployed [10]:

- The 868 MHz band in the European area with a raised-cosine-shaped Binary Phase Shift Keying (BPSK) modulation format. The ideal transmission range is approximately 1 km.
- The 915 MHz band in the North America and Pacific area with a raised-cosine-shaped Binary Phase Shift Keying (BPSK) modulation format. The ideal transmission range is approximately 1 km.
- The 2.4 Ghz ISM band with a half-sine-shaped Offset Quadrature Phase Shift Keying (O-QPSK) modulation format. The ideal transmission range is approximately 200 m.

Transmission is organized in frames which are designated as a Physical Protocol Data Unit (PPDU). There are four types of PPDUs: a beacon frame, a data frame, an ACK frame and a MAC command frame. All are formed with a Synchronization Header (SHR), a Physical Header (PHR) and a Physical Service Data Unit (PSDU) which is composed of a MAC Payload Data Unit (MPDU). The MPDU is composed of a MAC Header (MHR), a MAC Service Data Unit (MSDU) and a MAC Footer (MFR), except for the ACK frame who's MPDU is composed only of an MHR and an MFR.

To improve the transmission range IEEE 802.15.4 devices can self-organize into either star or multi-hop peer-to-peer topologies. Star topologies are preferable for small area low-latency applications whereas peer-to-peer topologies are better suited when a large area has to be covered and latency is not an issue. To support these topologies IEEE 802.15.4 defines two types of devices:

- *Full Function Device (FFD)*. FFDs contain the complete set of MAC services and can operate either as a WPAN coordinator, or as a simple device. FFDs are the only nodes allowed to form links with other devices.
- *Reduced Function Device (RFD)*. RFDs contain a subset of MAC services and can only operate as a network device.

### 2.3. ZigBee

The ZigBee architecture is made up of a set of blocks called layers. Each layer performs a specific set of services for the layer above. The IEEE 802.15.4-2004 standard defines the two lower layers: the PHY layer and the MAC sub-layer. On top of this the ZigBee standard defines the Network (NWK) layer and the application (APL) layer [13]. The NWK layer performs the following operations [14]:

- Configuring a new device. A new device can begin operation as a ZigBee coordinator or try to join an existing network.
- Starting a new network.
- Joining and leaving a network.
- NWK layer security.
- Routing frames to their destination. Only ZigBee coordinators and routers can relay messages.
- Discovering and maintaining routes.
- Discovering one-hop neighbors and storing one-hop neighbor information.
- Assigning addresses to devices joining the network. Only ZigBee coordinators and routers can assign addresses.

The APL layer consists of the application support sub-layer (APS), the ZigBee device objects (ZDO) and the manufacturer-defined application objects. Manufacturer-defined application objects use the application framework and share APS and security services with the ZDO. The APS provides an interface between the NWK layer and the rest of the APL layer components. The APS performs the following functions [14]:

- Maintain binding tables.
- Forward messages between bound devices.
- Manage group addresses.
- Map 64-bit IEEE address to 16-bit network address, and *vice versa*.
- Support reliable data transport.

While the ZDO performs the following functions:

- Define the role of the device (ZigBee coordinator, router, or device).
- Discover the devices on the network and their application. Initiate or respond to binding requests.
- Perform security-related tasks.

## 3. Building Automation Systems

Building automation system (BAS) is an umbrella term used to refer to a wide range of computerized building control systems. From special-purpose controllers to standalone remote stations, to larger systems including central computer stations and printers, a BAS comprises several subsystems which are connected in various ways to form a complete system. The system has to be designed and engineered around the building itself to serve the services systems for which it is intended. Consequently, although the component parts used may be identical, no two systems are the same, unless they are applied to identical buildings with identical services and identical uses [15].

Building services include HVAC systems, electrical systems, lighting systems, fire and security systems and lift systems. In industrial buildings they may also include the compressed air, steam and hot water systems used for the manufacturing process. A BAS may be used to monitor, control and manage all or just some of these services. In the following sub-sections we briefly describe some of the most commonly used communication standards in the BA industry.

### 3.1. BACnet

BACnet is a data communication protocol for BA and control networks. BACnet has been developed under the endorsement of the American Society of Heating, Refrigerating and Air-Conditioning Engineers (ASHRAE). It is an American national standard, a European standard, an ISO global standard and the national standard in more than 30 countries. It is the only open protocol that was designed originally for BA and supports functions such as scheduling, alarming and trending [15].

To achieve interoperability across a wide spectrum of equipment, the BACnet specification consists of three major parts. The first part describes a method for representing any type of BA equipment in a standard way. The second part defines messages that can be sent across a computer network to monitor and control such equipment. And the third part defines a set of acceptable LAN architectures that can be used to convey BACnet communications.

BACnet provides a standard way of representing the functions of any device, such as analogue and binary inputs and outputs, schedules, control loops, and alarms, by defining collections of related information called ‘objects’, each of which has a set of ‘properties’ that further characterize it. Each analogue input, for instance, is represented by a BACnet ‘analogue input object’ which has a set of standard properties such as present value, sensor type, location, alarm limits and so on. One of the object's most important properties is its identifier, a numerical name that allows BACnet to unambiguously access it. BACnet defines 25 standard object types. A BACnet device does not need to support all object types, but if an object type is supported, it must comply with the standard object model for that object type. BACnet employs the OSI Model as its reference model. The BACnet protocol defines a number of data link/physical layers, including Ethernet, BACnet/IP, Point-To-Point over RS-232, Master-Slave/Token-Passing over RS-485, ZigBee and LonTalk [15].

### 3.2. LonWorks

LON technology is used primarily for the decentralized processing of automation functions in room automation. LON can carry out monitoring, controlling and regulating functions for building services such as heating and ventilation systems. The focus of using LON technology at the automation level is not to decentralize individual functions, but to provide a standardized integrated bus system [16].

The heart of the LonWorks system is an integrated circuit called Neuron Chip. The Neuron Chip was developed by Echelon Corporation and comprises three processors that provide both communication and application processing capabilities. The Neuron Chip has to be used in conjunction with a transceiver for a specific medium; twisted pair, power line, radio frequency and fiber-optics. The most common medium employed in LonWorks is the twisted pair while the types of Neuron Chip most commonly used are the 3120 and the 3150 made by Toshiba and Cypress. The Neuron Chip type 3120 is usually used in simple devices that do not carry out complex functions, while the type 3150 is intended for more sophisticated applications [16]. Both types of Neuron Chips have three internal processors where each processor carries out different functions:

- CPU 1 is responsible for physical accessing the transmission medium. A network interface provides access to the transceiver. This represents layer one and two of the ISO/OSI model.
- CPU 2 is responsible for transmitting network variables and represents layers three to six of the ISO/OSI model.
- CPU 3 processes application programs but does not access the network. This is done by the other two CPUs.

The Neuron Chip 3120 has read-only memory which stores the LonTalk protocol, the Neuron operating system and the predefined operating routines for input/output (I/O) conditioning. On the other hand the Neuron Chip 3150 does not have internal ROM, but relies instead on external memory to store the aforementioned functions and the application program.

### 3.3. KNX

The KNX standard describes an open system concept for distributed home and building automation and control. KNX covers the full scope of home and building automation and control including lighting, shading, shutters and blinds, heating, ventilation, and air conditioning (HVAC), and remote meter reading. KNX emerged in 2002 as a merger between the European Installation Bus (EIB), Batibus and the European Home System (EHS) standards. The aim of this merger was to create a single European home and building electronic control systems standard [17,18].

The KNX standard defines the network protocol specification, rules and definitions of how a KNX system is managed and how devices implemented by different vendors have to behave to achieve internetworking. The KNX protocol stack is based on the ISO/OSI reference model. Since different communication media are supported, the KNX protocol stack is divided into a medium-dependent and medium-independent parts. The medium-dependent part of the protocol stack consists of the physical layer and the lower level of the data link layer (DL) while the medium-independent part includes the upper level of the DL, the network layer (NL), the transport layer (TL), and the application layer (AL).

For physical media KNX provides a choice of dedicated twisted-pair cabling, power line transmission, and RF communication. Communication over IP networks as a first class medium is currently in the draft stage.

The main KNX medium is the twisted-pair cabling variant known as KNX TP1. The single twisted pair carries the signal as well as 29VDC link power. Data is transferred in a character oriented manner via half-duplex bidirectional communication at a transmission rate of 9,600 bps. TP1 allows free topology wiring with cable lengths of up to 1,000 m per physical segment. Up to four segments can be concatenated using bridges, called line repeaters, forming a line. A line can contain up to 256 devices and up to 16 lines can be interconnected by main lines to form an area. Finally, up to 15 main lines can be interconnected by a common backbone line using routers called backbone couplers. Medium access on TP1 is controlled using CSMA with bit-wise arbitration on message priority and station address. Four priority levels are provided.

KNX Powerline 110 (PL110) uses the 230V/50Hz electrical power supply network for data and power transmission (in compliance with EN 50065-1). Half-duplex bidirectional communication is supported. KNX data is modulated using spread frequency shift keying (SFSK) with a center frequency of 110 kHz and a maximum transmission rate of 1,200 bps. The signal is injected between phase and neutral and is superimposed on the sinusoidal oscillation of the mains. Repeaters can be installed in three-phase networks if passive phase coupling is no sufficient. Medium access control is based on a slotted technique to reduce the probability of collisions: After the minimum silence period between two frames has elapsed, two time slots are reserved for pending high-priority transmissions, followed by seven more from which nodes with pending standard priority transmissions choose one at random as their starting time.

KNX RF uses a sub-band in the 868 MHz frequency band reserved for short-range devices by European regulatory bodies. Data is transmitted at a rate of 16.4 kbps using frequency shift keying (FSK) modulation and Manchester encoding. The KNX RF frame format is based on FT3 as specified in IEC 60870-5. To minimize hardware requirements KNX RF not only supports bidirectional communication but unidirectional transmit-only devices are also supported. KNX RF devices communicate peer-to-peer.

KNXnet/IP currently focuses on scenarios for enhancing central and/or remote management. The KNXnet/IP Core Services define the packet structure and methods required for discovery and self-description of a KNXnet/IP server and for setting up and maintaining a communication channel between the client and the server. KNXnet/IP specifies several service protocols. KNXnet/IP tunneling describes the point-to-point exchange of KNX data over the IP network. Its main purpose is to replace USB or EIA-232 connections between KNX network interfaces and PC workstations or servers by tunneling L_Data frames. Acknowledgements, sequence counters and a heartbeat mechanism are used to ensure robustness. KNXnet/IP routing is a point-to-multipoint protocol for routing messages between KNX lines over a high-speed IP backbone. KNXnet/IP routers send UDP/IP multicast messages to other KNXnet/IP routers on the same IP network, which in turn filter the messages according to their destination or group address and pass them to the native KNX segment. KNXnet/IP device management allows configuration and diagnostic of KNXnet/IP tunneling interfaces or routing devices via the IP network. While KNXnet/IP is designed for devices with one IP and one traditional KNX network interface, the upcoming KNX IP is also intended for end devices that are solely connected to the IP medium.

The DL defines services to send and receive frames over the network. Acknowledgement is available as an option for some network media. The two most important services defined are *L_Data* for peer-to-peer data frame transfer and *L_Poll_Data* for a master collecting data from slaves in a so-called polling group. Furthermore, the DL defines a generic addressing scheme that is common to all available network media.

The NL uses the services provided by the DL and offers four different NL services: a unicast service, a multicast service, a domain-wide broadcast service, and a system broadcast service. For all four services, the individual address of the sender is used as the source address while the destination address depends on the used service. Additionally the NL introduces a hop count which is decremented and examined by routers and repeaters to perform filtering based on the amount of elapsed hops of a packet.

The TL uses the NL services and adds a connection-oriented unicast service. Using this service a device can establish a reliable unicast connection to another device. The state machine used for this service implements an acknowledgement mechanism such that data packets are retransmitted in case of a negative of absent acknowledgement. The four NL services are also present unchanged in the TL.

The AL layer on top of the stack provides several AL services which can be broadly classified in two different classes: data process exchange *(process data communication)* and configuration and maintenance tasks *(management communication)*.

The internet working and application model specifies how data is represented in KNX and how it is accessible via the network. *Datapoints* associated with *functional blocks (FBs)* are a central concept in this model. A KNX datapoint may be related to a sensor value/actuator state or it may be a parameter that controls the behavior of the user application. The necessary association between datapoints is established via bindings. KNX also specifies the application interface that is presented to user applications for interacting with remote datapoints. Finally, profiles define which parts of the KNX specification have to be implemented and ensure interworking of devices with the same profile that are provided by different manufacturers [17,18].

## 4. Ambient Intelligence Frameworks

Due to the multidisciplinary nature of Ambient Intelligence systems and the diversity of problems approached, several frameworks have been developed. In this section we provide a brief description of some of these frameworks.

### 4.1. AMIGO

The AMIGO Project was a project developed by fifteen European companies and research organizations [19–24]. The project was concluded in 2008. The aim of the AMIGO Project was to develop a middleware that dynamically integrates heterogeneous systems to achieve interoperability between services and devices. Through this middleware devices such as home appliances, multimedia players that communicate through UPnP, and personal devices are connected in the home network to work in an interoperable way. This interoperability across different domains can also be extended across different homes and locations. AMIGO focused on four application domains: Personal Computing, Mobile Computing, Consumer Electronics and Home Automation.

The AMIGO architecture follows the paradigm of Service orientation, which allows developing software as services delivered and consumed on demand. Discovery mechanisms can be used to find and select the functionality that a client is looking for. The AMIGO architecture is formed by the following three main components:

1. *Base Middleware Layer*. The Base Middleware layer contains the functionality necessary to integrate a networked environment such as discovery, interoperability, security, quality of service, content distribution, billing, *etc.* This solution is based on the semantics that are used to communicate and discover available services and devices in the network, including those based on existing communication and discovery standards. Existing hardware and software and new services can be discovered and composed independently. Supported service discovery protocols are UPnP, SLP and WS-Discovery while supported service interaction protocols are SOAP and RMI.
  AMIGO's Middleware layer is further subdivided into the following components:
  - Interoperable Service Discovery and Interaction (SD&I) middleware.
  - Semantic service discovery.
  - Service composition, adaptation and execution.
  - Domotic Infrastructure.
  - Content discovery & adaptation.
  - Content storage & distribution.
  - Security.
  - Accounting and billing.
2. *Intelligent User Services Layer*. The Intelligent User Services layer contains the functionality needed to facilitate an ambient in-home network. This layer brokers between users and service providers, provides context information, combines multiple sources of information and makes pattern-based predictions. Information is tailored to user profiles and adapts to the user's situation and changes in context. The Intelligent User Services layer of the AMIGO Project is formed by the following components:
  - Context Management.
  - User modeling and profiling.
  - Awareness and notification.
  - User interface services.
  - User privacy.
3. *Programming and Deployment Framework*. The Programming and Deployment framework contains the resources necessary for programmers to develop AMIGO aware services. This NET/OSGi based programming framework contains libraries for service description, encryption, *etc.*

### 4.2. SOPRANO

SOPRANO's goal is to build an Ambient Assisted Living (AAL) system for elderly people with functional impairments [25–28]. SOPRANO seeks to provide flexible and personalized IT services that maximize the independence of elderly people with functional impairments and help them in retaining their dignity. Examples of such services are medication reminding, home automation, coping with increasing frailty, home safety and security, activity monitoring, keeping healthy and active, coping with cognitive ageing and forgetfulness, combating social isolation, and countering boredom.

The core of the SOPRANO system is the SOPRANO Ambient Middleware (SAM), which provides its intelligence by receiving user commands and data from sensors, enriches them semantically and provides appropriate reactions via actuators in the house. Planned sensors are, for example, smoke, temperature, door status, location of the user by Radar or RFID, its health status and so on. Planned actuators are speech synthesizers, digital TVs with avatars, device regulators (for switching devices on/off or modifying their behavior), emergency calls to a central and more. Additionally the more static context of the house and the user shall be taken into consideration when performing concrete actions. SAM can is divided into the following three main components:

1. *Context Manager*. The Context Manager constantly analyzes incoming sensor events as well as the status of networked devices and appliances and tries to deduce higher-level context information. The results of this analysis are context parameters change events of a high semantic level. Context is defined as the user's situation in terms of all the temporal, personal, organizational, environmental, and even global conditions surrounding the user at a certain instant in time. Examples are the user's current activity and long lasting profile, the user's environment like lighting and temperature, connectivity parameters and so on. The context parameters change events are the input of the Procedural Manager.
2. *Procedural Manager*. The Procedural Manager provides meaningful reactions to contextual changes or explicit user requests. Explicit user requests are handled by a subcomponent called the User Input Analyzer or UIA. By analyzing the new situation, the Procedural Manager compiles an abstract process description based on a repository of process templates. The abstract process description is a standard workflow description which contains abstract service requests instead of concrete service bindings. It can be parameterized with contextual variables, is based on pre-defined templates and is annotated with context-aware metadata. The procedural manager can obtain state information by invoking ad-hoc queries to the context manager. The result from this second step is a “plan of goals” which serves as input for the composer.
3. *Composer*. The Composer has two objectives. First it serves as SAM's interface to the “real world” through sensor information. All incoming and outgoing service calls are handled by the Composer. Second, it receives the “plan of goals” from the Procedural Manager, intelligently searches, compares, composes and parameterizes adequate concrete services in order carry out the process in a concrete manner and execute it through actuators.

### 4.3. MPOWER

The MPOWER project developed a middleware platform to support the rapid development and deployment of integrated services for the elderly and cognitively disabled [29–33]. MPOWER provides a domain specific architecture that facilitates interoperable, integrated, secure and standardized solutions that speed up the development process and create market possibilities for interested parties. Work was aligned with the standardization activities within the HL7 (Health Level 7) organization which was a key prerequisite for exchanging messages between different health providers.

The MPOWER SOA architecture consists of five layers:

1. *Application Layer*. Provides graphical user interfaces and serves as an entry point for using the services.
2. *Business Process Layer*. Defines the business rules of the applications that are created using the MPOWER middleware.
3. *Services Layer*. Contains the implementations of the services that are created within the MPOWER platform.
4. *Service Components Layer*. Service components expose the functionality of the components and databases in the Resource layer.
5. *Resource Layer*. This layer consists of existing custom built applications, such as databases storing patient-administrative, medication, and management information. Other relevant resources in this layer are smart sensors such as physiological monitoring devices, temperature sensors and burglar alarm systems.

The services provided are grouped into the following five categories:

1. *Information (Medical and Social) Services*. These services enable management of social information and events of the patient. For instance, these services handle information regarding patients' schedules, personal information, and social contacts.
2. *Interoperability Services*. These services enable interoperability of MPOWER platform with the other platforms that are relevant for health-care application. Primarily these services enable integration of medical services with existing medical systems of hospitals or particular state medication systems.
3. *Sensor Services*. Sensor services provide functionality to add, remove, and adjust devices as well as retrieve sensor information. The services expose both a management mechanism and data access thorough an easy to use and standardized interface.
4. *Contextual Services*. These are services related to the monitoring and management of the context of a patient. For instance, these services provide information about location of the patient in his house. The context services are realized through a set of sensors and actuators located in the patient's premises.
5. *Security Services*. Ensure sufficient protection for any of the MPOWER enabled services when they are used. This implies that security middleware is orthogonal to the other services in a way that is an implicit part of each service, ensuring a satisfactory security level of any combination of services in the MPOWER platform.

### 4.4. universAAL

The universAAL project seeks to combine the advantages and strengths of still ongoing or already finished research projects to create a universally applicable AAL platform [34–38]. universAAL reuses components and concepts from the AMIGO, GENESYS, OASIS, MPOWER, PERSONA and SOPRANO projects. universAAL seeks to achieve its goals through the development of an open source middleware targeted towards health, home automation, entertainment, and energy efficiency applications and services. universAAL will also provide reference use cases and a tool chain for extending platform capabilities. Support for the creation of AAL services and applications will be provided through the universAAL Developer Depot and uStore. Based on the GENESYS and PERSONA projects universAAL makes use of a layered design. The layers that make up the universAAL platform are the following:

1. *Middleware*. The Middleware extends the native system layer of the different physical nodes participating in an AAL system. It hides the distribution of these nodes as well as the possible heterogeneity of their native system layers. Additionally this layer acts as a container for the integration of the components from the above layers and facilitates the communication among them.
2. *Generic Platform Services*. The Generic Platform Services layer provides basic platform services like context management, service management, and a framework for supporting complex user interactions.
3. *AAL Platform Plug-Ins*. On this layer special platform services can be introduced to extend the basic functionality of the framework. This might be needed in case high-level services have specific demands on, for example, data-mining of context reasoning.
4. *AAL Applications and Services*. The AAL Applications and Services layer encapsulates all applications and services that directly provide support and assistance to the end user.

The list of frameworks presented in this section is by no means exhaustive. The frameworks presented here are some of the largest and best funded projects but many others, such as DOBS [39], GAIA [40], One.world [41], PCOM [42], and TinySEP [43] exist.

## 5. Control Systems and Function Blocks

A control system is a device or set of devices to manage, command, direct or regulate the behavior of other devices or systems. Although control systems of various types date back to antiquity, a more formal analysis of the field began with a dynamics analysis of the centrifugal governor, conducted by Maxwell in 1868 entitled “On Governors” [4].

Many industrial control systems fall into one of two categories, either based on traditional distributed control systems (DCSs) or on programmable logic controllers (PLCs). DCSs like the ones commonly found in petrochemical plants and refineries are structured around a few large processors that provide supervisory control and data acquisition communicating via local networks with controllers, instruments, sensors and actuators located out in the plant. These types of systems may have both discrete instruments and out-stations with clusters of instruments with local controllers. In a DCS the main supervisory control comes from one or more of the central processors while instruments positioned out in the plant typically provide local closed loop control such as PID control.

On the other hand, many machine control and production processes such as those found in automotive production lines have generally been designed using PLCs. A large PLC system will generally have a number of PLCs communicating via one or more proprietary high-speed networks. PLCs will generally be connected to a large number of input and output (I/O) signals for handling sensors and actuators. In some cases discrete instruments, for example for temperature and pressure control, are also connected to PLCs.

With both design approaches systems have tended to be developed by writing large monolithic software packages which are generally difficult to re-use in new applications and are notably difficult to integrate with each other. The data and functions of one application are not easily available to other applications even if they are written in the same programming language and are running in the same machine.

In order to promote the development of flexible solutions the concept of function blocks was introduced in industrial systems. A function block is a robust, re-usable software component. The function block is an abstract model representing a function that can be implemented by software and/or hardware. A function block can provide a software solution to a small problem such as the control of a valve, or control a major unit of a plant, such as a complete production line. Function blocks allow industrial algorithms to be encapsulated in a form that can be readily understood and applied by people who are not software specialists. Each block has a defined set of input parameters, which are read by the internal algorithm when it executes. The results from the algorithm are written to the block's outputs. Complete applications can be built from networks of function blocks formed by interconnecting block inputs and outputs [44,45].

### 5.1. IEC 61499 Standard

The function block concept was standardized for programmable logic controllers in the International Electrotechnical Commission's IEC 61131 standard [46]. To extend the concept of function block outside the realm of PLCs the IEC 61499 standard was developed. The IEC 61499 built upon the function block concept defined in IEC 61131-3 to develop a generic standard that can also be applied in other industrial sectors, such as in building management systems [47].

IEC 61499 defines a general model and methodology for describing function blocks in a format that is independent of implementation. It allows a system to be defined in terms of logically connected function blocks that run on different processing resources. IEC 61499 provides terminology, models and concepts to allow the implementation of a function block oriented distributed control system to be described in an unambiguous and formal manner. Having a formal and standard approach to describing systems allows such systems to be validated, compared and understood [44,45].

While previous standards employ a data or signal based communication among the constructs and assume a cyclic execution, IEC 61499 introduces an event driven approach of interaction among the function blocks. This means that algorithms are executed only if an input event activates the block. As a result functions are separated from the execution control. Writing a program with function blocks involves drawing a network of function blocks and allocating them to devices for execution [44,45,48].

As the trend to use component based software continues, it is foreseen that industrial controllers and instruments will either provide function blocks as part of the device firmware or provide function block libraries from which blocks can be selected and downloaded. System design will become the process of software component selection, configuration and interconnection, just as much of electronic hardware design is now primarily concerned with the selection and interconnection of IC chips. In a function block world, the system designer's main focus is to take standard proven encapsulated functionality and link it together in the quickest and most intuitive way possible. The use of function blocks is nearer to the mind-set of the industrial system designer who is familiar with connecting physical devices together in different ways to provide a particular system solution [44,45].

**Figure 1.**
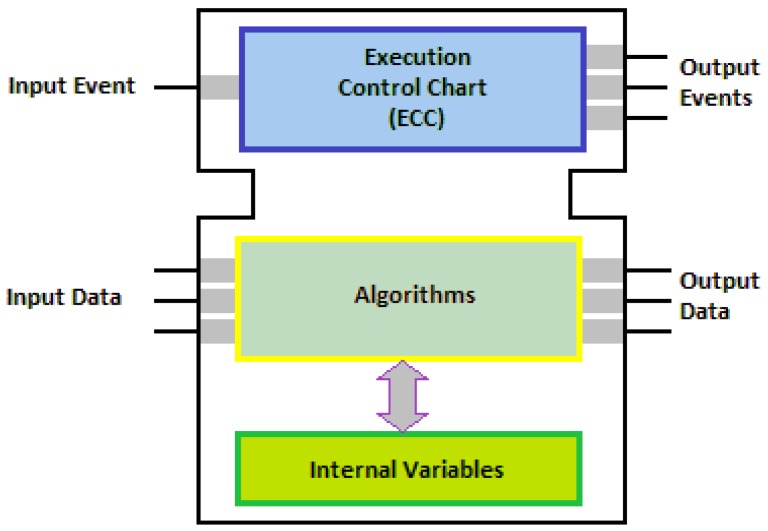
IEC 61499 function block.

The Function Block (FB), the basic construct of IEC61499, consists of a head and a body as shown in Figure 1. The head is connected to the event flows and the body to the data flows, while the functionality of the function block is provided by means of algorithms, which process inputs and internal data and generate output data. The sequencing of algorithm invocations is defined in the FB type specification using a variant of state charts called Execution Control Chart (ECC).

An ECC consists of EC states, EC transitions and EC actions. An EC state may have zero or more associated EC actions, except from the initial state that shall have no associated EC actions. An EC action may have an associated algorithm and an event that will be issued after the execution of the algorithm. EC transitions are directed links that represent the transition of the FB instance from one state to another. An EC transition has an associated Boolean expression that may contain event inputs, data inputs, and internal variables. As soon as this expression becomes true the EC transition fires [48].

**Figure 2.**
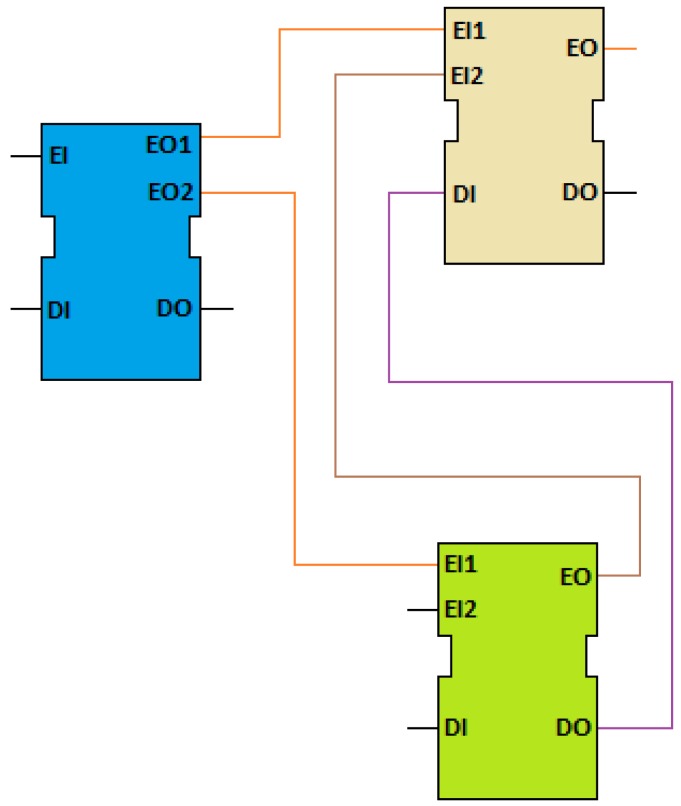
Function block network.

FB instances are interconnected to form a Function Block Network (FBN), as shown in Figure 2. A FBN may be executed on a single device or on a network of interconnected devices. A device may contain zero or more resources, where a resource is considered to be a functional unit, contained in a device which has independent control of its operation and may be created, configured, parameterized, started-up, deleted, *etc.*, without affecting other resources within a device. The event connections and behavior of every single block completely determines the behavior of the network. An application in IEC 61499 is composed by one or more FBNs.

**Figure 3.**
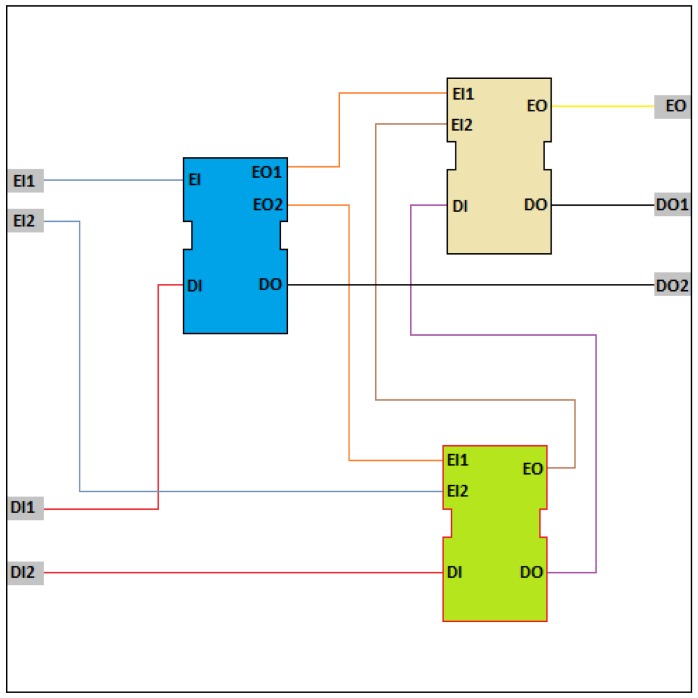
Composite function block.

Composite function blocks (Figure 3) provide a means for building up more complex blocks from basic and other smaller composite blocks in a hierarchical fashion.

The type definition for a composite function block contains declarations of function block instances of selected types that are linked by data and event connections. The standard regards blocks that are used within a composite block as component function blocks. The data connections between component blocks define the transfer of data values between block outputs to inputs while the event connections define the order of execution of algorithms within the blocks. A composite function block is just a container for a network of other function blocks. The container as such performs no specific actions except for setting input-output variables and for the activities of its components. The network can include basic and composite function block types [44,45].

## 6. FunBlocks Framework Description

FunBlocks is an event-driven, minimalist, modular framework for the development of Ambient Intelligence systems [3,49]. FunBlocks approaches the development of AmI systems from the point of view of distributed control system using the function block abstraction described in the IEC 61499 standard. FunBlocks also makes use of a publish/subscribe, store and forward interaction scheme which is a well-established mechanism for the development of loosely coupled event-based distributed systems [50,51]. Developing an AmI system using the FunBlocks framework consists of developing or reusing function blocks which provide the desired functionality, and afterwards joining these blocks through the services provided by the framework.

### 6.1. FunBlocks Components

**Figure 4.**
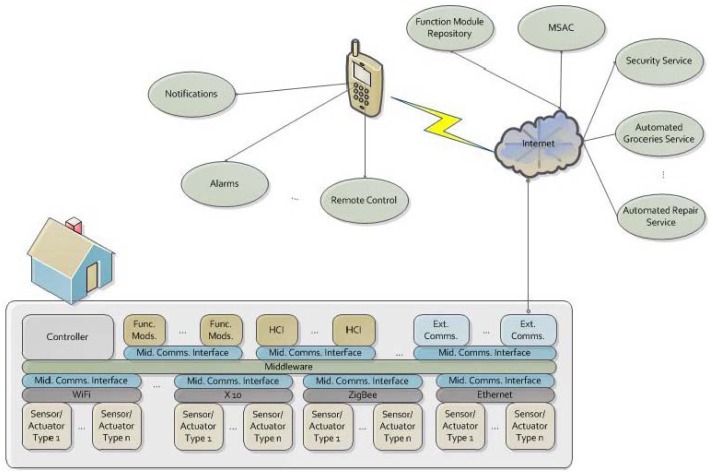
FunBlocks diagram.

To begin with an overall view of FunBlocks, in this section we provide a brief description of the components that constitute the framework. In later sections we will describe with more detail some of these components. FunBlocks is formed by the following components (Figure 4):

- Controller (CTRL). The main functions performed by the Controller are the following:
  - Maintains information of the SAs and modules installed in the system and makes this information available to new modules.
  - Notifies modules when new SAs are installed.
  - Performs communication with external components, such as Automated Repair Services.
  - Prevents the installation of modules which could generate conflicts, such as multiple Gesture Recognition modules in the same area.
- Middleware (MDLW). The Middleware provides the communication mechanism through which the components of the system interact. FunBlocks middleware also performs supervisory activities generating failure events in case a component exceeds its Maximum Event Interval without generating any events. This middleware can be expanded through the use of Middleware Communications Interface components.
- Middleware Communications Interface (MCI). In order to provide AmI systems with the flexibility of integrating a wide array of different devices and modules, the MDLW can be extended through Middleware Communications Interfaces. Some of these MCIs may be formed both by software and hardware components to allow operation with different device types, such as X 10 sensors and actuators.
- Sensors/Actuators (SAs). As previously described SAs provide a means to obtain information from and interact with the environment.
- Human/Computer Interaction (HCI). HCI components allow users to perform explicit interaction with the AmI system. As explained later on HCIs are considered as a special type of SAs. HCIs include touch panel interfaces and computing devices such as laptops or smart phones.
- Module and Sensor/Actuator Catalog (MSAC). The Module and Sensor/Actuator Catalog allows the storage and retrieval of Module and Sensor/Actuator Description (MSAD) records. These records contain a description of the services provided by the component, for example a door entry sensor, a camera, or a speech recognition module, and also contain a description of the components requirements. The MSAC is meant to be housed in a location external to the AmI system such as a manufacturers Internet site, however, once a record is obtained from the MSAC it is stored locally, which means that a connection to the MSAC has to be established only when adding or removing components to the system.
- Function Modules (FM). Function Modules communicate with sensors either directly or through the MDLW depending upon the data encoding format used. FMs perform specialized services such as speech recognition and media streaming.
- Function Module Repositories (FMRs). FMRs house the modules themselves and allow downloading modules for installation on the system.
- External Communications Modules (ECM). These modules provide a link to external communications networks such as the Internet or telephone services.

### 6.2. Sensors and Actuators

Through sensors AmI systems gather information about physical environmental parameters such as heat, humidity, temperature, ambient light intensity, *etc.* and information about the activities currently being performed by people. Through actuators, AmI systems adjust environmental parameters in order to assist users in their activities. As a result, sensors and actuators (SAs) are the fundamental means of interaction of an AmI system. In order to allow FunBlocks to handle many different sensors and actuators in an easy and consistent manner, we broadly classify SAs into three major categories:

1. *Binary SAs (BSAs)*. As the name implies, binary sensors and actuators have two states, such as a smoke detector or a garage door open-close actuator.
2. *Multi-valued SAs (MVSAs)*. Multi-valued sensors and actuators can provide more than two states. Examples of these types of sensors are temperature sensors and shade positioning actuators.
3. *Special Function SAs (SFSAs)*. Sensors and actuators in this category provide a richer and more complex means of interaction with the environment. Examples of devices in this category are Kinect devices, keyboard-display interfaces, and speech synthesis units.

Each category of sensors contains different types of sensors. BSAs include smoke detectors, presence detectors, gas leakage detectors, *etc.* MVSAs include water level sensors, temperature sensors, humidity sensors, light intensity sensors, *etc.* Our design does not assume a priori knowledge of all the types of SAs. We simply assume that any device which allows an AmI system to interact with the environment will fall into one of the previously described categories and that a module to handle the data from, or to send commands to, the SA is available.

**Figure 5.**
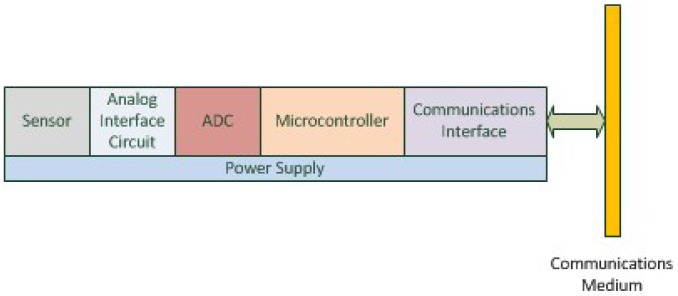
Smart sensor block diagram.

A smart transducer is obtained by combining a transducer, an analog interface circuit, an analog to digital and/or digital to analog converter, a microcontroller, a power supply and a communications interface [52]. We consider that all SAs handled by FunBlocks are smart transducers (see Figure 5) [3].

This results in an extremely flexible design. Any SA communications protocol can be integrated into the system through an MCI and entire foreign systems can be treated as SAs if wrapped by an appropriate gateway. Consider for example the case of an AmI system developed with FunBlocks that has to operate with an existing security system. Depending on the facilities provided by the security system, it can be used as a simple intruder alarm by FunBlocks or, with the development of an appropriate gateway, it can be activated and deactivated through FunBlocks.

An important concern during the design of FunBlocks was that of reliability. Devices will fail. Depending on the purpose of the device its failure can lead to a potentially hazardous or even lethal situation. Consider for example the case of a smoke detector or LP gas detector. Failure of such a device can lead to injury or death in case of a fire or a gas leak and therefore should be notified immediately. As a result, these types of devices must be queried frequently enough to insure that they are “alive”.

The use of wireless media such as 802.11 and 802.15.4 coupled with the use of batteries as a power supply has become rather common for sensors in recent years. Wireless sensors include a transceiver as part of their communications interface. This transceiver is the part of the sensor responsible for the consumption of most of the energy and therefore the transceiver is the component that drains more current from the battery in wireless battery powered sensors [10]. Both transmit and receive states are highly energy consuming and as a result the sensor should remain in these states for the shortest possible percentage of time.

This leads to a contradictory situation in which sensors must be queried frequently in order to assure their responsiveness and simultaneously should only transmit when a change in the environment occurs to avoid draining their batteries quickly. To solve this, the SAs and other components of FunBlocks must generate an event periodically either as a result of their operation or as a form of notification. BSAs generate an event when a change in state occurs, for example, when a door is opened or when a person enters an area. If a time interval has been exceeded without any change of state, then the BSA must generate a heartbeat event. The exact time interval between heartbeat or state change events must be configurable and depends on the type of parameter being sensed. MVSAs on the other hand generate events periodically. The exact time interval between the events generated by an MVSA must be configurable and depends on the type of parameter being sensed. For example, a time interval of 5 or 10 min between events for a temperature sensing SA might be adequate since the temperature variation in a room will not be very severe during this time interval. Both BSAs and MVSAs must also be able to return their current value when polled and, if possible, will also generate an event in case of a malfunction.

SFSAs must also generate periodic events. Although failure of certain types of SFSAs such as display-keyboard units are unlikely to represent a threatening situation, detection of such a failure is important in order for users to have confidence in AmI systems.

Frequently environments are divided into different units each with individual control requirements. Consider for example a typical house. A house is divided into distinct areas for sleeping, socializing, cooking, *etc.*, and each of these areas have to be controlled separately. To provide a means for segmenting an environment into controllable units FunBlocks groups SAs into areas. An area is simply a way to group sensors in a functionally related form. For example, if the lights in a room are to be turned on when presence is detected, the presence sensor and the light actuator have to be assigned to the same area.

In order to uniformly handle different types of sensors FunBlocks establishes a minimum set of parameters which can be obtained from all sensors regardless of its type. This minimum set of parameters is enough to allow an AmI system to perform the following operations:

- *Presence*. An AmI system must be able to detect the removal of a sensor. This allows components whose operation relies on data from the sensor to take adequate action.
- *Failure*. Sensors with self-diagnosing capabilities must be able to notify the AmI system in the event of a failure.
- *Grouping*. SAs require some form of grouping scheme. This is required in order to assure proper evaluation of stimulus by the AmI system and that a correspondence can be established between stimulus and action.
- *Interpretation*. An AmI system must be able to adequately interpret data coming from sensors. In particular there must be an unequivocal way to relate sensor readings with ambient parameters.

To achieve these operations FunBlocks uses a simple message scheme in which event notification and data are transmitted simultaneously in the same message. In this scheme both the event and the data associated with the event are transmitted simultaneously through XML event messages which contain the following fields:

- SENSOR ID. This is a unique identifier assigned to each sensor by the system when the device is installed. This allows to keep track of sensors and to generate a system malfunction event in case a sensor stops generating events.
- AREA. As mentioned before, SAs are assigned to areas. This field indicates the area this sensor belongs to.
- TYPE. A numerical identifier assigned to this class of devices by the manufacturer or system integrator.
- DATA. This is the data obtained by this sensor.

With this simple message format an AmI system developed with FunBlocks can perform the operations described previously:

- *Presence*. Presence is detected by event messages generated by the sensors. Since each sensor can be uniquely identified through its SENSOR ID, if a sensor has failed to generate an event after its assigned maximum time interval then the AmI system can assume that the sensor has failed. For sensors that are either critical or unable to report failures, absence or failure of a sensor should be treated in the same manner. This is particularly important for critical sensors since the inability of the AmI system to receive sensor data can lead to potentially hazardous situations.
- *Failure*. Sensor failure is detected either through absence of sensor messages or by an explicit message from sensors with self-diagnosing capabilities.
- *Grouping*. Grouping is achieved by means of the AREA field. All sensors which have the same AREA identifier belong to the same group.
- *Interpretation*. Interpretation of sensor data is achieved through the TYPE field. The value in the TYPE field corresponds to the identifier of the MSAD record of the sensor. The MSAD record contains the information necessary to interpret the sensor's data.

Care should be exercised when implementing the TYPE and DATA fields in a particular system. If sensors made by different manufacturers share the same TYPE identifier then these sensors should be interchangeable. Since the structure of the DATA field is given by the sensor's MSAD record, the DATA field can be customized for any sensor. However, if a high degree of customization is chosen for a particular TYPE of sensor then the use of very sensor specific FMs will be required. This results in a loss of flexibility.

### 6.3. Middleware

A middleware is a software layer which allows the components of a distributed system to interact [53]. A common type of middleware is the publish/subscribe middleware. In a publish/subscribe middleware producers publish information on a software bus (an event manager) and consumers subscribe to the information they want to receive from that bus. This information is typically denoted by the term event and the act of delivering it by the term notification [50,51]. The strength of this event-based interaction style lies in the full decoupling in time, space and synchronization between publishers and subscribers [50].

Due to the need of integrating different devices such as SAs, HCIs, existing HA and BA systems, *etc.*, AmI systems are inherently distributed systems. As a result AmI systems middleware has been a topic of great interest. The full decoupling in time, space and synchronization achieved through the use of the event driven paradigm makes this type of middleware an excellent candidate to integrate the components of an AmI system. For example, in [54–56] the use of a publish/subscribe event-based mechanism for AmI systems called FamiWare is described. FamiWare is a middleware family based on a reduced implementation of the OMG Data Distribution Service (DDS) standard. Through the use of the Software Product Line (SPL) engineering approach, FamiWare provides customized device- and application-specific configurations of the middleware.

Other approaches to integrating the components of an AmI system include the use of distributed object middleware. PicoObjects provide a toolset for the automated generation of code which allows embedded systems to interact with object-oriented middleware such as CORBA [57–60].

**Figure 6.**
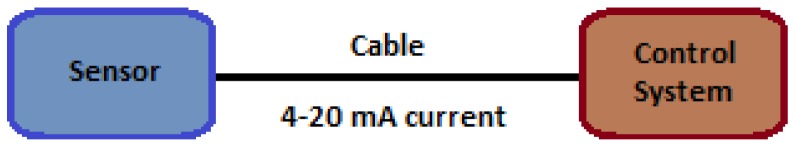
4–20 mA current-loop.

In order to provide reliable operation, an AmI system must be able to supervise its components and warn users in the event of a malfunction. The need to supervise the components of a system is nothing new and has frequently arisen in diverse settings. A frequent solution to the problem has been to introduce supervisory capabilities to the communications scheme employed by a system. Two examples of this type of solution are the pneumatic control systems used in early building automation and the popular 4–20 mA current-loop (Figure 6) used in industrial measurement and control systems.

In early building automation systems hoses with compressed air were used to transmit information from, and power to, the sensors, actuators and controllers in the system. 3–15 psi was the modulation standard, with 3 psi being a live zero and 15 psi for 100% [61]. Any pressure below 3 psi is a dead zero and an alarm condition. The 4–20 mA current-loop works in exactly the same way with 4 mA being a live zero and 20 mA 100%. Any current above or below this range signals a malfunction either in the device or in the wiring [61,62].

The role of a publish/subscribe system is to permit the exchange of events between producers and consumers in an asynchronous manner. Asynchrony can be implemented by having producers send messages to a specific entity that stores them and forwards them to consumers [50]. In FunBlocks, we employ this store and forward entity to additionally perform component supervision tasks.

Event messages arriving from producers are received by the Message Receiver (MR) where they are stamped with a set of configurable Time-To-Live (TTL) fields, one TTL field for each consumer. Afterwards the messages are inserted into the Message Queue (MQ). Messages are retrieved from the MQ by the Message Dispatcher (MD) on a FIFO basis and delivered to the consumers of the event. If the message is delivered successfully then it is marked as delivered to the consumer, otherwise the TTL for that particular consumer is decremented by one. If one of the message's TTL fields reaches zero then no further attempts will be made to deliver the message to the corresponding consumer, and the message will be marked as undeliverable for that consumer.

The Component Supervisor (CS) scans the MQ for the arrival of event messages from the system's components and for messages marked as undeliverable. If a component has exceeded its maximum time interval between events the CS generates a failure event for the consumers that have subscribed to failure events from this component. On the other hand, if the CS finds a message marked as undeliverable it will also generate a failure event notifying the failure of the consumer and will mark the message as processed for that particular consumer. Finally, the Garbage Collector (GC) deletes all messages from the MQ that have been marked as delivered or processed for all of its consumers.

This mechanism allows FunBlocks to supervise the modules that form an AmI system without the need of a separate heartbeat event. Furthermore, by notifying other modules in the system in case one of them fails, an AmI system might take corrective actions in case of a module failure. Consider for example the failure of a smoke sensor. If a smoke sensor fails an AmI system might shutdown the main gas valve and part of the electrical system to reduce any fire hazards. Simultaneously, the system might use a temperature sensor so that if the temperature in a room rises beyond a given threshold then the fire alarm is sounded as a precautionary measure.

### 6.4. Function Modules and Function Modules Repository

When an application is installed in certain operating systems, such as many Linux distributions, a sequence of actions takes place:

1. A package which contains the application is downloaded. The package also contains a list of packages required for this package to operate and an installation procedure.
2. The system verifies that each of the required packages listed in the requirements is installed. If any are missing the package is downloaded and installed using this same procedure.
3. Once the requirements are installed the installation procedure is carried out and the package is installed.

This installation method can be viewed as a form of composing an application from a group of modules.

In IEC 61499 an application is a network of function blocks. To compose an application with IEC 61499 the function blocks that constitute the application have to be installed in different devices and the event and data links between the modules have to be established. FunBlocks performs this action through the Middleware, the Module and Sensor/Actuator Catalog and the Function Module Repository. Through its MSAD record in the MSAC an application (which is itself an FM) describes the FMs required for it to operate. The connections between the FMs that form the application are established through the publish/subscribe method provided by the MDLW and any missing FMs can be obtained from one or more Functional Module Repositories.

This scheme allows applications to be composed with minimum effort on behalf of the AmI system user or integrator. Furthermore, as new FMs are created as part of the development process of a new AmI system or as a result of a research project, such FMs can be reused either as part of commercial AmI systems or in new research projects.

### 6.5. Component Interaction

As mentioned in the introduction an Ambient Intelligence system can be viewed as a particular case of an event driven distributed control system. Specifically we consider that any type of activity that is carried out by the AmI system is triggered by an action performed by the user, by a change in the environment or by an internal time event.

The CTRL, MDLW, MSAC and FMRs form the basic platform upon which an Ambient Intelligent system is integrated. The CTRL and MDLW by themselves do not contain any AmI functions. AmI functions are performed by the FMs and SAs.

When a new FM is installed, the CTRL obtains the MSAD for the FM from the MSAC. MSAD records describe, among other things, the SA types required by this FM to operate, the events generated and the services provided by this FM. For example, a Facial Recognition FM requires a Presence BSA and a Video Stream SFSA, and generates USERID-INAREA events. An Emergency Assistance Module requires some type of hazard sensor, e.g., a Smoke Detector BSA or Gas Leakage BSA and some type of warning SA, such as a Siren BSA. When a new FM is installed it obtains from the CTRL a list of the areas, SAs and other FMs installed on the system. Next it subscribes to events generated by the types of SAs it can handle and, in case further configuration is required, notifies the user. The notification and configuration is carried out through the CTRL on the HCI employed by the user to request the installation of the FM.

The use of Function Modules to provide services, Function Module Repositories to house the modules, and the Module and Sensor/Actuator Catalog to obtain the services/requirements of each component gives AmI systems developed with FunBlocks a high degree of adaptability for different scenarios. Modules such as Voice Recognition, Facial Recognition, Gesture Recognition, Media Streaming, Message Retrieval, Automated Grocery Request, *etc.* can be installed depending on the user's needs. These modules will usually be installed without the need of specialized personnel, notifying the user and automatically ordering or installing any additional SAs and modules required for their operation in much the same way as modern operating systems, such as Ubuntu Linux, can install new software. The need for such a system customization mechanism has been described by Bernheim *et al.* and is being introduced into other frameworks such as universAAL.

### 6.6. Usage Scenario

To illustrate the flexibility offered by FunBlocks we describe a simple usage scenario. Consider a user who lives alone and has a simple Ambient Intelligence System consisting of a Presence BSA, a Message Retrieval Module (MRM), and a Display Enabled Mirror HCI (DEM HCI) near the entrance of his home. Every morning when he is leaving for work the MRM obtains his messages and displays them on the DEM HCI. Such a setup was easily hooked up by the user by assigning the Presence BSA and the DEM HCI to the same area, and configuring the MRM to activate from Monday to Friday between 7:30 and 8:00 am. The MRM was designed to respond to user presence events and display messages on any HCI available in the same area from which the presence event originated.

Later on the user's girlfriend moves in and she also wants to have her messages displayed before she leaves for work. The user instructs the CTRL through a Smartphone HCI to install a Facial Recognition Module (FaRM). The CTRL obtains the MSAD record for the FaRM and determines that a camera is required. Through the MSAC, the CTRL determines that there is a camera accessory for the DEM HCI, warns the user through the Smartphone HCI that the FaRM requires a camera and requests authorization to order the camera accessory. The user grants authorization and the CTRL orders the camera.

When the camera arrives, the user snaps it in the accessory receptacle of the DEM HCI and the camera is detected. The CTRL then proceeds to download and install the FaRM from the manufacturers FMR. During the installation procedure the FaRM requests that nicknames of the users be entered and pictures be taken using the camera accessory. Afterwards it notifies the CTRL of its ability to generate USERID-INAREA events for each user. The CTRL notifies the MRM of the new events and the module requests that the information required to retrieve the messages of each user be entered. After the information is entered, the MRM unsubscribes to presence events and subscribes to USERID-INAREA events.

This simple usage scenario illustrates some key benefits of the FunBlocks framework:

- User interaction is kept to a minimum and does not require any specialized skills.
- By notifying the modules of the changes in the system, a module can adapt its behavior to the new resources available.
- Through the use of the MSAC the system was able to assist the user in obtaining the missing components needed to satisfy the requirements of the new system.

## 7. Features Comparison

Ambient Intelligence is a currently a field of intense research (see for example [63,64]) but as of yet there is not an AmI systems development framework that enjoys widespread use. Unfortunately, nowadays most of the effort in AmI applications concentrates itself on developing the most sophisticated applications in order to corner the market, and the profits [65]. Consequently, current proposals tend to be rather complex, which means that a research or development team has to invest significant effort in order to be able to use the framework. Ease of use has been one of our main goals during the development of FunBlocks.

The problems that have been approached by the developers of different frameworks are varied, and as a result the characteristics of the frameworks are also diverse. In [66] Becker describes a set of system and service qualities that an Ambient Assisted Living system should possess. This set of system and service qualities are valid in general for any Ambient Intelligence system. The system and service qualities that should be achieved by AmI systems are the following:

1. *Affordability*. The services and the required technical installation and resources must be affordable.
2. *Usability and User Experience*. If the system requires some explicit interaction with the user, the system should perform the interaction accordingly with the user's capabilities and the user should have a positive experience during such interaction.*3*.
3. *Suitability*. The services provided by the system must meet the demands of the user and the user must benefit from the services, otherwise acceptance of the system will decline over time.
4. *Dependability*. The system must be robust against misuse, errors, hardware component crashes and shortage of resources. Furthermore the system must guarantee a minimum of privacy and security for the users. Finally the system must be safe to use and not pose a risk to its users.
5. *Adaptivity*. Systems must be able to adapt themselves at runtime. Systems must monitor themselves, users and the environment and based on the information gathered perform one of the following:
  - *Self-Configuration*. Integrate dynamically new components and remove existing components not required any more.
  - *Self-Healing*. Denotes the ability of detecting component problems and taking appropriate measures.
  - *Self-Optimization*. The ability of the system to adapt its algorithmic behavior to application requirements changes.
  - *Self-Protection*. Denotes the ability of the system to protect itself against misuse.
6. *Extensibility*. In order to adapt to new demands the system must be able to extend itself with new devices and services.
7. *Resource Efficiency*. Available resources such as processors, memory, communications bandwidth and energy have to be used as efficiently as possible.
8. *Heterogeneity*. The system will typically consist of several subsystems provided by different manufacturers.

In Table 1 we provide a brief list of some of the features of the frameworks described previously along with the corresponding features of FunBlocks. The criteria used for the comparison are the following:

1. *Purpose*. As mentioned before frameworks tend to be targeted toward the solution of specific problems such as AAL.
2. *Architecture*. Current frameworks are based on different paradigms being SOA the most popular. The architecture selected for the framework will have a strong impact on its system and service qualities. Particularly affordability, adaptivity, extensibility, and heterogeneity are influenced by the choice of architecture.
3. *Platform*. The platform selected for the implementation of the system will have a direct impact on affordability and heterogeneity and is therefore an important consideration in the selection of a framework.
4. *Component Supervision*. Component supervision is an important feature which helps improve the dependability of a system.
5. *Module Repository*. The use of a Module Repository allows end user customization of the system hence improving usability and user experience.
6. *Sensor Handling*. AmI systems have to interact with a vast array of existing sensor technologies. The choice of the sensor handling scheme in the framework will have a direct impact on the affordability and heterogeneity of the system.

## 8. Conclusions and Future Work

FunBlocks provides an extremely flexible framework for the development of Ambient Intelligence systems. Contrary to other AmI frameworks, FunBlocks makes no assumption about the types of environments or devices that can be handled in an AmI system. As a result FunBlocks can be used transparently with existing systems such as home/building automation systems, security systems, *etc.*

By considering AmI systems as a type of distributed control systems, and by making use of the IEC 61499 function block abstraction, FunBlocks can be used to develop AmI systems which can be customized to a wide range of different usage scenarios. FunBlocks is also language and hardware independent, which means that the framework can be used with a vast variety of devices ranging from 8 bit microcontrollers to servers. Language and hardware independence along with the function block abstraction also promotes the reuse of research efforts.

The use of a middleware with supervising capabilities allows AmI systems developed with FunBlocks to monitor the operational state of their components and, whenever possible, to take corrective actions in case of component failure. This feature helps to improve the dependability of an AmI system. Since the middleware makes use of a publish/subscribe paradigm with a store and forward entity for component interaction it can easily be reused in other event-based systems which require component monitoring [67].

There is still much work to do developing FunBlocks. The current design of FunBlocks does not allow the use of redundancy in components such as the controller and the message queue. As a result these components represent single points of failure. The use of redundant controllers and redundant message queues must be addressed in order to provide a higher degree of reliability. Once the issue of redundancy has been addressed self-healing and service composition capabilities must be introduced in FunBlocks. This is particularly important for wide area systems where communications links could fail leading to a fragmented system. It would be desirable for each fragment to reconfigure itself into an independent AmI system perhaps with limited capabilities.

Finally further testing is required. Current tests with FunBlocks have been carried out with small scale implementations mainly testing different parts of the framework. The next stage in the development of FunBlocks is the implementation of full-scale systems. We will be focusing on the implementation of previously developed systems to test and further refine the features offered by FunBlocks. Simultaneously implementing such full-scale systems in FunBlocks and in some of the previously mentioned platforms will allow a comparison of the features offered by the diverse proposed approaches. Once the framework reaches a minimum state of stability it should be release under some license, preferably open source, to allow other interested parties to develop AmI systems based on FunBlocks.

## Figures and Tables

**Figure 7.**
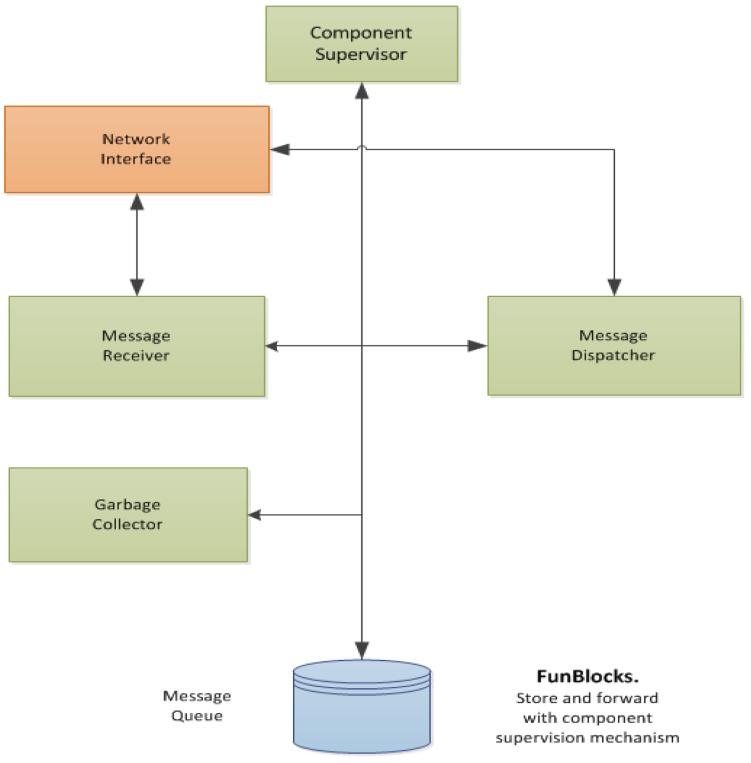
FunBlocks middleware.

**Table 1.** Framework features.

	**Amigo**	**MPOWER**	**SOPRANO**	**universAAL**	**FunBlocks**
**Purpose**	Dynamic Interoperability	AAL	AAL	AAL	General Purpose
**Architecture**	SOA	SOA	SOA	SOA	Function Block
**Platform**	NET/OSGi	Web Services, HTTP, SOAP	OSGi	OSGi	Platform Independent
**Component Supervision**	No	No	Yes	No	Yes
**Module Repository**	No	No	No	Yes	Yes
**Sensor Handling**	Through wrappers	Network enabled	As OSGi bundles	Networked enabled	Native prot. support
